# Analysis on the energetics, magnetism and electronic properties in a 45° ZnO grain boundary doped with Gd

**DOI:** 10.1039/c8ra00985f

**Published:** 2018-04-13

**Authors:** Assa Aravindh Sasikala Devi, Iman S. Roqan

**Affiliations:** King Abdullah University of Science and Technology, Physical Science and Engineering (PSE) Division Thuwal Saudi Arabia iman.roqan@kaust.edu.sa

## Abstract

The structural stability and magnetic properties of a grain boundary (GB) formed by aligning two ZnO single crystals oriented at an angle of 45° is investigated by density functional theory, using generalized gradient approximation (GGA) and taking the *U* parameter into consideration for the 4*f* impurity states. We found that the GB is stable with no dangling bonds and inter-granular structures. The stability of defects such as Gd substituted to the Zn site (Gd_Zn_), Zn vacancy (V_Zn_) and O vacancy (V_O_) as well as defect complexes Gd_Zn_–Gd_Zn_, Gd_Zn_–V_Zn_, and Gd_Zn_–V_O_ are analyzed using formation energy calculations. It is found that Gd_Zn_–Gd_Zn_ clusters prefers to form at the GB. The spin polarization at the Gd_Zn_ sites is too localized and the exchange coupling energy is insufficient to overcome the thermal fluctuations. However, we show that the presence of V_Zn_ increases the hybridization between p orbitals of O as well as d orbitals of Zn, which can assist in increasing the magnetic polarization of the system. This work advances the understanding of the ferromagnetism in Gd-doped ZnO, indicating that Gd clustering at the GB is not likely to contribute to the ferromagnetism.

## Abstract 2

The structural stability and magnetic properties of a grain boundary (GB) formed by two ZnO single crystals oriented at 45° is investigated by density functional theory, using generalized gradient approximation (GGA) with Hubbard parameter (*U*).

## Graphical abstract

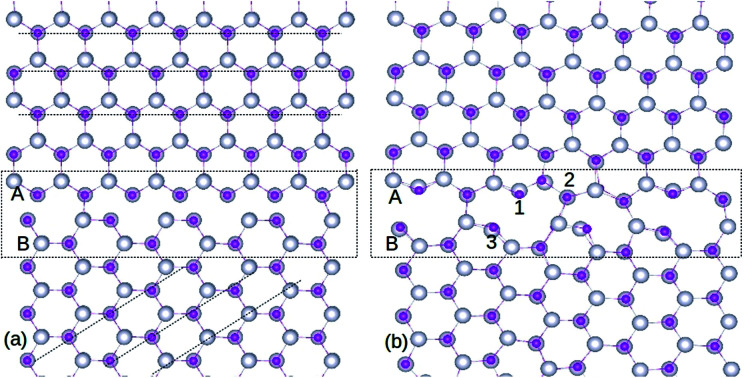

## I. Introduction

Dilute magnetic semiconductors (DMSs) have emerged as potential candidates for spintronic applications, owing to their semiconducting and ferromagnetic characteristics.^1^ While GaAs and GaN based DMS were the subject of most prior investigations in this field,^2^ the focus has recently shifted to ZnO, as it exhibits room temperature ferromagnetism (RTFM) in presence of transition metals (TMs)^3^ and rare earth (RE) elements.^4–7,19^ The existence of intrinsic defects, such as dislocations, grain boundaries (GBs), and vacancies in ZnO also assists in inducing magnetic properties,^8^ and the ferromagnetic coupling strength is increased when defect-dopant complexes are present in the material.^3^ In particular, ZnO GB can play a role in the ferromagnetism (FM) of ZnO. Both stoichiometric and non-stoichiometric GBs have been found to significantly affect the properties of ZnO materials, such as producing distinct occupied and unoccupied levels, with changes in the coordination and concentration of O atoms, which in turn produces varistor effect in the undoped conditions.^9^ Recently, we have also demonstrated FM in ZnO GB in presence of cation vacancies,^8^ wherein FM originates from the localized holes forming triplet states, thus giving rise to a stable magnetic ground state.^10^ This magnetic phenomenon originates from the p band, rather than the d band (in which case it is referred to as the d^0^FM.^11,12^. However, the formation energy of cation vacancies in bulk ZnO is extremely high, rendering the existence of RTFM almost impossible, thereby requiring another mechanism to strengthen the magnetism originating from vacancies, which can be achieved by several means, including impurity doping.^12^

Interesting ferromagnetic and electronic properties are observed in bulk and nanostructured DMS materials doped with RE elements.^2,6,13,14^ RTFM in RE-doped ZnO thin films and nanostructures has been observed. Magnetic moments as high as 7 and 12 μ_B_ per atom were obtained in ZnO nanowires and thin films, respectively,^7^ doped with Gd owing to the strong exchange interaction between 4f and 6s electrons.^15^ ZnO single crystals implanted with Gd atoms were also reported to produce saturation magnetic moments reaching 1.81 μ_B_ per Gd atom,^16^ whereas *in situ* Gd doped ZnO produced 5–12.35 μ_B_ per Gd.^7,19^. Moreover, while Gd is a promising dopant for obtaining FM in the dilute limit (≤3.5%),^17^ at elevated concentrations in ZnO thin films, it was shown to exhibit paramagnetism.^18^ The ferromagnetic behavior of Gd-doped ZnO is attributed to the existence of different defects. For instance, in extant studies, FM in Gd-doped ZnO thin films was ascribed to the formation of Gd complexes with oxygen deficiency-related defects *via* a spin-split defect band formed near the Fermi level.^7,19^ These findings suggested that, by controlling the concentration of Gd defects and vacancies, long-range FM can be obtained.^7,19^ However, the origin of the FM in Gd-doped ZnO is not well-understood as intrinsic defects can play a significant role in mediating/inducing and stabilizing the exchange interactions in Gd-doped ZnO. Many researchers posit that FM may originate from dopant clusters in ZnO GB defects.^20,21^ However, thus far, the effect of GB defects in mediating/inducing FM in Gd-doped ZnO has not been investigated in detail.

Here, we report a systematic density functional theory (DFT) study of Gd-doped ZnO GB, in presence of intrinsic point defects, such as Zn vacancy (V_Zn_) and O vacancy (V_O_). We primarily aim to find if FM can be obtained in presence of Gd atoms at the GB, as well as to investigate the possibility of Gd clustering by performing formation energy calculations. The impact of Gd clusters and Gd-defect complexes on long range ferromagnetic interactions in the material is analyzed using electronic structure calculations.

## II. Computational methodology

The simulations were carried out using plane wave based DFT method, as implemented in Vienna Abinitio Simulation Package (VASP).^22,23^ We have used projected augmented wave (PAW) based pseudo potentials, while the exchange and correlations were described using the Perdew–Burke–Ernzerhof (PBE) functional.^24,25^ It is well known that owing to the strong electronic correlations of d electrons, standard DFT cannot accurately describe the electronic structure of ZnO. To overcome this, the Hubbard *U* parameters^26^ are employed along with generalized gradient approximation (GGA), such that *U*_Zn_ = 7, *J*_Zn_ = 1, *U*_Gd_ = 7.4 and *J*_Gd_ = 0.5 eV, in line with the Dudarev's approach.^27^ It is well known that sufficient plane waves must be included in the basis set for accuracy and to account for this, kinetic energy cut-off of 400 eV was used. To standardize the procedure, we calculated the unit cell parameters for bulk ZnO and these optimized values were employed to construct the GB. The calculated unit cell parameters for bulk ZnO, *a*_0_ = *b*_0_ = 3.32 Å and *c*_0_ = 5.219 Å, are used to construct the GB supercell such that *a* = 7 × *a*_0_, *b* = √3 × 3 × *b*_0_; and *c* = *c*_0_. The lattice constants *a* and *c* based on the experiments mostly vary in the 3.2475–3.2501 Å and 5.2042–5.2075 Å range, respectively.^28^ Lattice parameter values of *a*_0_ = *b*_0_ = 3.2507 Å; *c*_0_ = 5.2083 Å have been obtained by conducting X-ray diffraction experiments, whereas energy dispersive X-ray diffraction (EDXD) results showed *a*_0_ = *b*_0_ = 3.2498 Å; *c*_0_ = 5.2066.^28^ Hence, our calculated wurtzite ZnO lattice parameters are in good agreement with corresponding experimental values, attained using different techniques. The Brillouin zone sampling was carried out using a Monkhorst pack *k* mesh of 1 × 1 × 6 and the GB supercell was optimized within energy and force tolerances of 0.001 eV and 0.001 eV Å^−1^, respectively. For the density of states calculations, we have used a slightly larger *k* mesh. All the calculations reported in this work were performed with the inclusion of spin-polarization.

**Fig. 1:**
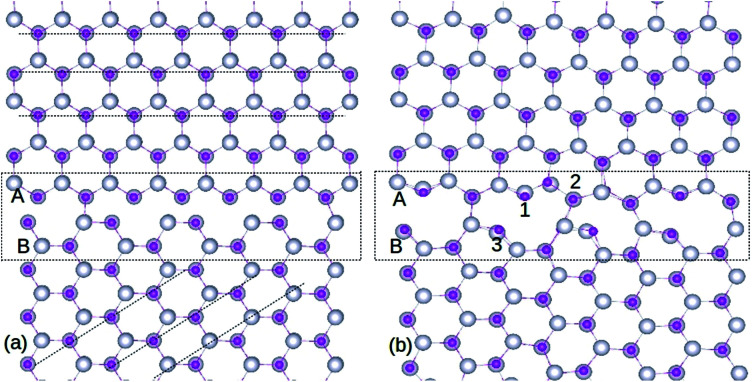
Structure of ZnO GB, before (a) and after (b) optimization. The silver and pink colored spheres represent Zn and O atoms respectively. The doping locations of Gd atoms are marked as 1, 2 and 3 in (b). A and B represent the two single crystals of ZnO used to create the GB supercell. The atomic rings formed after optimization can be seen from (b). The amorphous GB region is indicated by the rectangle, and dashed lines are guide to the eye.

We constructed the GB using two ZnO supercells oriented at a 45° angle relative to each other, each containing 148 atoms.^3,8^ The preliminary ZnO super cell with 296 atoms is presented in Fig. 1(a), and the optimized supercell is shown in Fig. 1(b). After optimization, it is evident that Zn and O atoms are well bonded at the GB with no inter-granular structures. The structural units at the GB consist of 8- and 10-membered atomic rings, whereas bulks ZnO possess a six-fold coordinated open channel. The atomic rings at the GB consist of Zn and O atoms with 4- and 3-fold coordination, in contrast to bulk ZnO in which only 4-fold coordination exists. This repeated ring pattern establishes the periodic nature of the atomic arrangements at the GB.

It is important to note that the relaxed GB is stable without any inter-granular structures or wrong (Zn–Zn or O–O) bonds (see Fig. 1(b)), which is the bench mark for assessing the ZnO GB stability as reported in extant literature.^29^ However, atomic relaxation induces an alteration of ∼0.05 Å in the Zn–O bond lengths in the 8- and 10-membered rings, compared to those measured in the bulk-like region. Hence, the organization of periodic units along the GB efficiently minimizes the strain in its vicinity. Moreover, while the GB structure will be repeated due to periodicity, this is not going to alter the ZnO system properties, as the symmetry will be maintained.

## III. Results and discussion

The presence of GBs in materials creates diverse effects with respect to their structural, magnetic and electronic properties compared to the bulk, as these are greatly affected by the atomic structure. The varied atomic co-ordination manifesting as under- or over-coordination of atoms in a GB creates dangling bonds that can facilitate formation of native defects and segregation of dopants.^8^ As GBs with different angles affects the material properties in different ways,^30^ ZnO GBs with low and high angles^31^ characterized by short and long periodicity were investigated previously. For example, Sato *et al.*^32^ obtained a wide variety of GB atomic structures in ZnO solely by changing the relative orientation of the GB planes. Since an uncontrollable wide range of GB angles may be obtained under experimental conditions, in this work, we investigated a 45° GB as an example in order to gain better understanding of ZnO GB effects. It should be noted that, as the properties of low- and high-angle GBs may vary, the 45° GB chosen for this study represents only one of the many angles that occur in materials. In other words, in this work, we aimed to investigate the effect of one such GB (as an example) on the magnetic and electronic properties of Gd-doped ZnO compared to bulk Gd-doped single crystal ZnO without GB.^33^ In this work, we focus on the magnetic phenomena occurring in ZnO due to the combined effects of GB defects and Gd impurities in the presence of V_Zn_ and V_O_ (complex defects). We have previously shown that the presence of native point defects and dopant-defect complexes at the GB produces significant FM coupling in Zn.^3,8^ The inculcation of Gd into the ZnO GB is particularly interesting due to the fact that Gd was observed to enhance ferromagnetic coupling in presence of defects both in bulk and in nano structured DMS materials.^12,13^

We have previously estimated the energetics of V_Zn_ and V_O_ in GB of ZnO in ref. 6, using the defect formation energy (*E*^f^(*D*)) equation,

1 *E*^f^(*D*) = *E*(*D*) − *E*(ZnO) + *n*_i_*μ*_i_ + *qE*_F_

where *E*(*D*) and *E*(ZnO) indicate the total energy of GB containing point defect and that of pure GB respectively, whereas *n*_i_ and *μ* denote the number and the chemical potential of Zn/O atoms removed to create the point defect. The chemical potential is dependent on experimental growth conditions and calculated from the total energy of O_2_ molecule, elemental Zn and Gd. Here *q* represents the electron charge, whereas *E*_F_ denotes the Fermi energy.

Substitution of Gd atoms at the Zn sites is carried out at the GB in such a way that one Gd atom corresponds to a doping concentration of 0.33%. The Gd_Zn_ sites are depicted in Fig. 1(b) and the formation energy (*E*^f^) of Gd_Zn_ is calculated using the equation,

2 *E*^f^(Gd_Zn_) = *En*Gd_Zn_ − *E*(ZnO) + *nμ*(Zn) − *mμ*(Gd) + *qE*_F_

where the first and second terms indicate the total energy of GB containing a Gd atom and pure GB respectively, while *n* and *m* represent the number of Zn/Gd atoms removed/introduced to the GB, and *μ* is the chemical potential. Since we are not considering different charge states in these calculations (*q* = 0), the last term is eliminated from both eqn (1) and (2).

Three different locations are considered for the Gd atoms (Fig. 1(b)) and the resultant *E*^f^ values are shown in Table 1. The selection of these atoms is based on their dissimilar bonding arrangements in the GB; hence, they also represent other atoms in the nearby atomic ring. We find that, after optimization, Gd atoms remain stable in the atomic rings at the GB. The position labeled 3 in Fig. 1(b) exhibits the lowest *E*^f^ (−4.55 eV) among the three locations, as shown in Table 1. This demonstrates the advantage of the reduced coordination number at this neck site, which in turn assists in breaking the atomic bonds effectively. The small energy differences (<0.5 eV) among the three locations signify that the Gd atoms can easily substitute the Zn sites at the GB due to the comparable bonding arrangements of the cationic sites, irrespective of their spatial location. On the other hand, a Gd atom substituted for the Zn atom in the bulk-like region is less stable (*E*^f^ = −3.28 eV). In addition, the data presented in Table 1 shows that Gd impurity is a more preferred defect, compared to isolated V_O_ and V_Zn_ at the GB, indicating that GBs play a significant role in increasing the miscibility of Gd dopants in ZnO. Increasing the Gd dopant concentration leads to greater stability of point defects at the GB, as their *E*^f^s decrease significantly (>2 eV), as shown in Table 1. Domingos *et al.*^34^ found that the V_Zn_ formation energy can be reduced at the ZnO GB in presence of metallic impurities. The point defects stability in the GB can be due to the distortion introduced by the Gd in replacing a Zn site, as Gd atoms are of a much larger size compared to Zn.

**Table 1:** The formation energy, *E*^f^ (in eV) of Gd_Zn_ with and without point defects at the ZnO GB

Configuration	*E* ^f^ (eV)
Gd_Zn_-1	−4.55
Gd_Zn_-2	−4.16
Gd_Zn_-3	−4.42
Gd_Zn_–V_O_	1.56
Gd_Zn_–V_Zn_	−1.00
V_O_	4.1[from ref. 2]
V_Zn_	3.8[from ref. 2]
Gd_Zn_-in-bulk	−3.28

To study the aggregation tendency of Gd dopants, we have introduced another Gd_Zn_ at the GB. Table 2 presents the calculated *E*^f^ values as a function of the separation distance (*D*) between two Gd atoms, indicating that the *E*^f^ increases with their relative distance. Hence, forming a Gd_Zn_–Gd_Zn_ dimer is most probable at a nearest neighbor position compared to well-separated impurities, which assists in Gd segregation at the ZnO GB. This segregation occurs as, owing to its larger size relative to Zn, Gd can induce greater local strain in the vicinity, which attracts other Gd atoms to populate nearby sites. The occurrence of crystal strain as a result of Gd doping in ZnO thin films, leading to a reduction in the *c*-lattice parameter, was demonstrated through X-ray diffraction and Rutherford backscattering measurements. Such strain can arise due to the introduction of point defects during Gd doping, mainly the presence of V_Zn_.^5^ A similar effect has been observed in extant studies of Pr-doped ZnO GBs.^35^

**Table 2:** The formation energy, *E*^f^ (in eV) of Gd_Zn_–Gd_Zn_, at the ZnO GB for different *D* values (in Å)

*D* (Å)	*E* ^f^ (eV)
2.54	−9.87
3.2	−9.50
3.31	−9.22
6.96	−8.56
10.14	−8.10

The presence of more than one Gd atom also allows calculation of the magnetic exchange interactions. We find that the magnetic moment of Gd is very localized and induces no spin polarization to the nearby atoms, which is also observed in Gd-doped bulk ZnO in experiments.^21^ Hence, it is important to examine the effect of the presence of Gd-complex with intrinsic point defects on the FM in Gd-doped ZnO GB, as this will aid in the understanding of the impact of the GB defects on the magnetic properties. We have previously shown that V_Zn_ is energetically more favorable in undoped ZnO GB than in the bulk-like region and is also preferred at the GB compared to V_O_.^8^ These observations suggest that the presence of vacancies at the GB may enable the formation of vacancy–dopant complexes. To confirm this postulate, we have introduced V_O_ and V_Zn_ at the GB in presence of Gd_Zn_–Gd_Zn_ dimer in the nearest neighbor position and the corresponding binding energies are presented in Table 2. Since the *E*^f^ of point defects is lower in presence of Gd at the GB, the occurrence of vacancy–dopant complexes is preferred energetically.

**Fig. 2:**
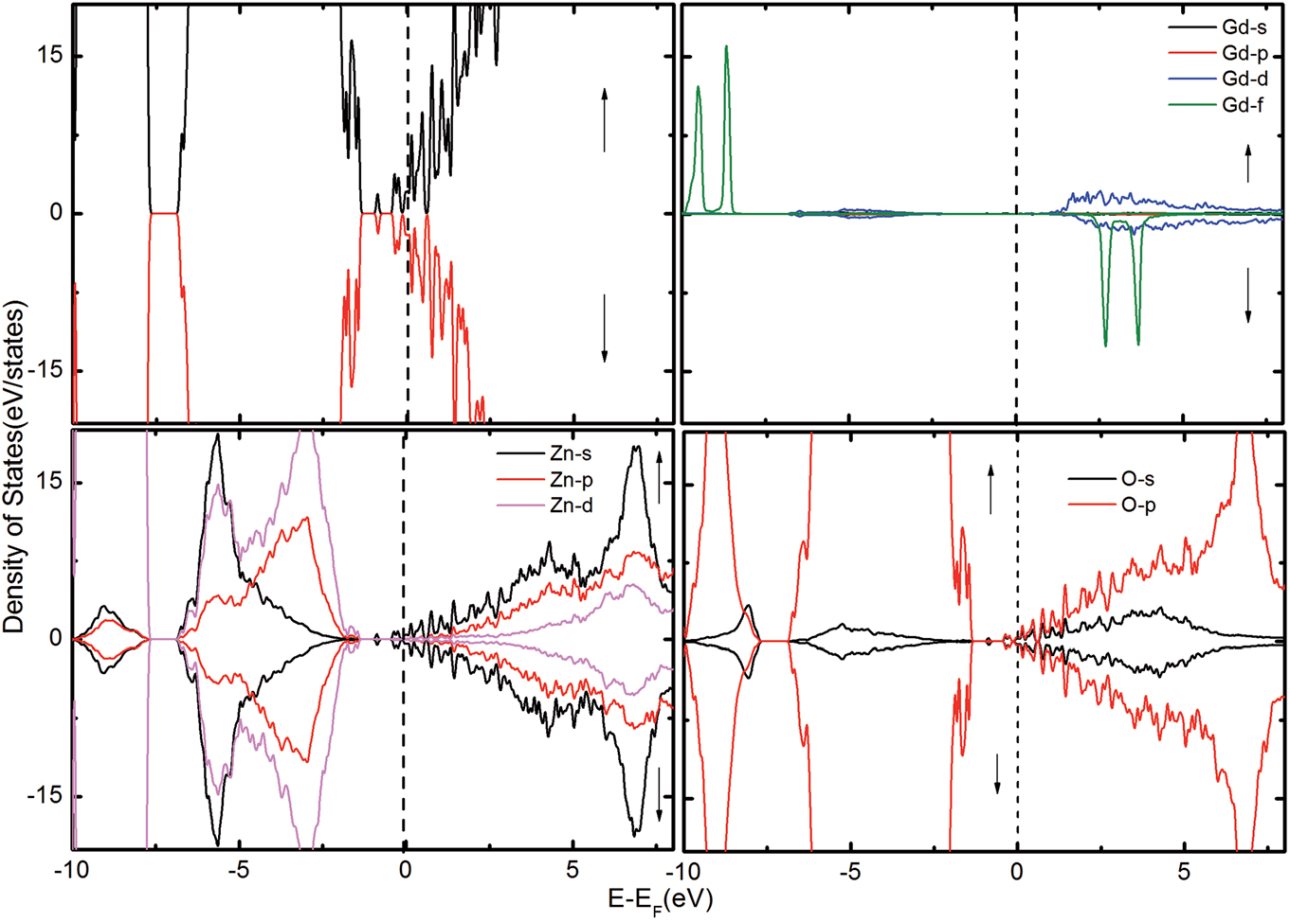
The TDOS of the ZnO supercell containing the GB along with Gd atoms doped at the GB. The corresponding PDOS of Gd, Zn and O atoms are also shown.

**Fig. 3:**
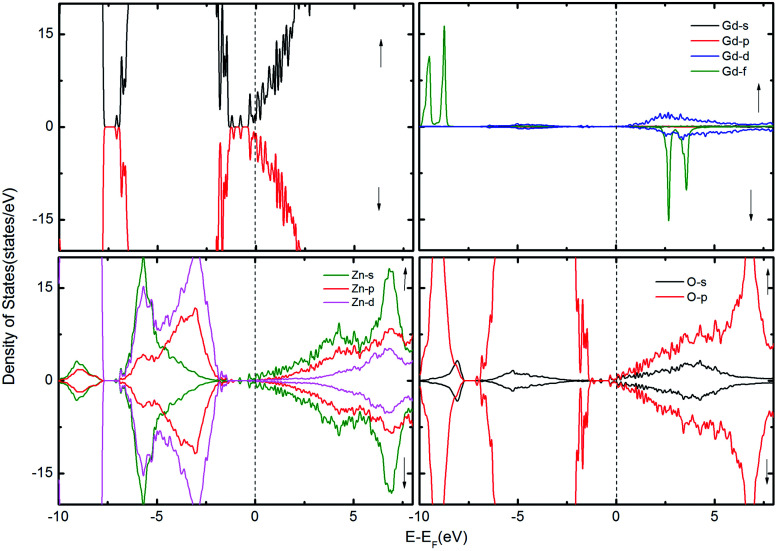
The TDOS of the ZnO supercell containing the GB along with Gd atoms in the presence of V_O_. The corresponding PDOS of Gd, Zn and O atoms are also shown.

**Fig. 4:**
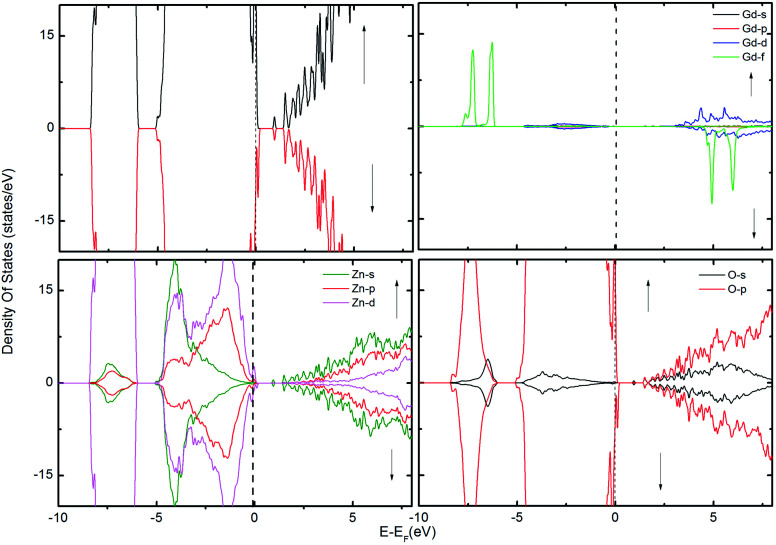
The TDOS of the ZnO supercell containing the GB along with Gd atoms in the presence of V_Zn_. The corresponding PDOS of Gd, Zn and O atoms are also shown.

To gain further insight into the magnetic mechanism, the density of states (DOS) was calculated for the ZnO supercell containing the GB. This is an established procedure for showing the changes in band gap induced by the effects of co-existence of external dopants and GB in the ZnO supercell.^30,36^ Three different scenarios were considered when two Gd atoms are in the nearest neighbor positions, namely (i) two Gd atoms are introduced at the GB without intrinsic defects, as shown in Fig. 2; (ii) Gd atoms with *V*_O_ (Fig. 3); and (iii) with V_Zn_ (Fig. 4). The total density of states (TDOS) and the projected density of states (PDOS) of Zn, O and Gd atoms are shown in Fig. 2–4. The TDOS of Gd (without any vacancies) indicates the absence of FM, as shown in Fig. 2. No spin splitting in the states at the *E*_F_ is observed, signifying that no carriers are available for facilitating the exchange interactions. The spin-up and spin-down peaks of Gd f states are very localized and are at a considerable distance from the band edge, as well as from *E*_F_, causing no magnetic exchange. Since the band edge states at the top/bottom of the valence band (VB)/conduction band (CB) respectively, are instrumental in determining the magnetic exchange, the positioning of *E*_F_ at the valence band maximum (VBM) or conduction band minimum (CBM) indicates that hole/electron density is inadequate for originating magnetic exchange interactions.^37^ Hence, the supercell exhibits paramagnetic behavior in presence of Gd_Zn_ at the otherwise defect-free GB.

Further, the ferromagnetic exchange coupling energy (Δ*E*) for a pair of Gd atoms is calculated as the energy difference between the energies of antiparallel *E*_AFM_ and parallel *E*_FM_ spin configurations such that,

3 Δ*E* = *E*_AFM_ – *E*_FM_

A positive energy difference implies favorable FM configuration, while negative value indicates an AFM configuration. For the nearest neighbor Gd_Zn_–Gd_Zn_ pair, at a distance of 2.54 Å, Δ*E* of 5 meV is obtained, which is too low to enable sustainable long-range RTFM in the system, since a minimum of 30 meV is required to establish magnetic coupling against thermal fluctuations. Previously, carrier doping has been utilized to successfully establish FM in ZnO bulk and nanostructures in presence of dopants.^3,13,38^ Through controlled doping, Kittilstved *et al.*^38^ showed that FM in Co-doped ZnO system is dependent upon carrier concentration. These results were subsequently confirmed by Lany *et al.*,^39^ who demonstrated that electron doping introduces strong FM coupling between the dopant ions. In Gd-implanted ZnO, Potzger *et al.*^16^ revealed an increase in the saturation magnetization owing to the presence of charge carriers released upon annealing, which was later supported by the results reported by Ungureanu *et al.*^15^

To examine the possibility of carrier enhanced FM, we have introduced 2V_O_ and 2V_Zn_, which effectively induces electron and hole carriers to the system respectively. Our findings revealed that V_O_ and hence electron carriers do not assist in increasing the exchange coupling, as evident from Fig. 3. Though Shi *et al.*^40^ have achieved an increase in FM in the presence of electron carrier in single crystal Gd-doped zinc blende ZnO, our results pertaining to FM in ZnO GB derived from the wurtzite structure fail to indicate any promising FM. Nevertheless, the introduction of hole carriers by the V_Zn_ in the vicinity of Gd_Zn_–Gd_Zn_ increases the coupling energy to ∼15 meV. Even though this exchange energy is insufficient for sustaining FM against thermal fluctuations at room temperature – (25 meV), induction of a greater density of hole carriers can strengthen magnetic interactions in the system.

## IV. Conclusions

We have used DFT with the GGA + U approximation to study the energetics, magnetism and electronic properties of Gd-doped ZnO 45° GB in presence of point defects. We have found that the Gd atoms are more stable at the GB, compared to the bulk-like region, and prefer to aggregate. The formation energy calculations showed that Gd doping stabilizes the native point defects such as V_O_ and V_Zn_. Our findings indicate that V_Zn_ can assist in inducing magnetic polarization to the GB, because in presence of Gd_Zn_–V_Zn_ complex the p orbitals of O hybridize with the d orbitals of Zn and Gd. However, since the spin polarization emanating from Gd_Zn_ atoms is highly localized, large concentration of V_Zn_ is needed to strengthen the magnetic interactions in the GB. Our results demonstrate that the Gd defects alone (generalized to other RE impurities) at such GB do not contribute significantly to the FM observed experimentally in Gd-doped ZnO.

## Conflicts of interest

There are no conflicts of interests to declare.

## Supplementary Material
