# Correction: Analysis on the energetics, magnetism and electronic properties in a 45° ZnO grain boundary doped with Gd

**DOI:** 10.1039/c9ra90001b

**Published:** 2019-01-18

**Authors:** Assa Aravindh Sasikala Devi, Iman S. Roqan

**Affiliations:** King Abdullah University of Science and Technology, Physical Science and Engineering (PSE) Division Thuwal Saudi Arabia iman.roqan@kaust.edu.sa

## Abstract

Correction for ‘Analysis on the energetics, magnetism and electronic properties in a 45° ZnO grain boundary doped with Gd’ by Assa Aravindh Sasikala Devi *et al.*, *RSC Adv.*, 2018, **8**, 13850–13856.

The authors regret that an incorrect grant number was shown in the Acknowledgements section of the published article. The corrected section should read:

We acknowledge financial support from the General Directorate of Research Grants, أت-34-405, from King Abdul-Aziz City of Science and Technology (KACST), Kingdom of Saudi Arabia.

The Royal Society of Chemistry apologises for these errors and any consequent inconvenience to authors and readers.

## Supplementary Material

